# The association between medical students’ lifestyles and their attitudes towards preventive counseling in different countries

**DOI:** 10.1186/s12889-015-2458-y

**Published:** 2015-11-14

**Authors:** Yan Yu, Yuxuan Yang, Zhifang Li, Bo Zhou, Yi Zhao, Shen Yuan, Ruijuan Zhang, Matthew Sebranek, Lennert Veerman, Mu Li, Enying Gong, Shu Chen, Wenjie Ma, Liping Huang, KaWing Cho, Stephen Leeder, Lijing Yan

**Affiliations:** Xi’an Jiaotong University School of Medicine, No.76#, West Yanta Road, Xi’an, 710061 Shaanxi China; Changzhi Medical College, Changzhi, Shanxi Province China; First Hospital of China Medical University, Shenyang, Liaoning Province China; Ningxia Medical University, Yinchuan, Ningxia Hui Autonomous Region China; Duke Global Health Institutesss, Duke University, Durham, USA; School of Public Health, the University of Queensland, St Lucia, Brisbane Australia; School of Public Health, the University of Sydney, Sydney, Australia; Children’s Hospital Los Angeles, Los Angeles, CA USA; Menzies Centre for Health Policy, School of Public Health, the University of Sydney, Sydney, Australia

## Abstract

**Background:**

Preventive counselling is an effective approach to reducing the prevalence of non-communicable diseases. Studies have shown that there is a positive association between healthy behaviors of Colombian medical students and favorable attitudes towards preventive counselling. However, there is limited research that explores this relationship in different countries. The current study aimed to determine how the health behaviors of medical students from China, U.S., and Australia, are associated with attitudes towards preventive counseling.

**Methods:**

Students from five Chinese medical schools, Duke University in the U.S., and the University of Queensland in Australia, completed a 32-item, self-reported online survey. The survey was used to examine the prevalence of healthy behaviors and their association with attitudes towards preventive counseling. The target sample size was 150 students from each grade, or 450 students in total from different medical universities. Logistic regression analyses were used to assess the association between health behaviors and attitudes towards preventive counseling, stratified by grade and adjusted by gender.

**Results:**

A positive association was found between healthy behaviors and attitudes towards preventive counseling for all medical students. There are significant differences among medical students’ self-reported health behaviors and their attitudes towards preventive counselling from three different countries (*P* < 0.05). Chinese medical students were more positive in stress control (OR > 1) and more passive in limiting their smoking and alcohol behaviors compared to medical students in Duke University. However, compared to medical students in University of Queensland, five Chinese medical students were more passive in stress control (OR < 1).

**Conclusion:**

Based on the finding that healthy behaviors are positively related to favorable attitudes towards preventative counselling, medical students should adopt targeted courses and training in preventive counseling and develop healthy lifestyles.

## Background

Non-communicable diseases (NCDs) - mainly cancers, cardiovascular diseases, chronic respiratory diseases, and diabetes—are the leading cause of death worldwide, accounting for almost 70 % of deaths in the world in 2013 [1]. It is predicted that NCDs will contribute to 73 % of the total deaths and 60 % of the disease burden worldwide by year 2020 based on the current trends [2]. Unhealthy lifestyles such as physical inactivity, smoking and excessive consumption of alcohol are known risk factors for NCDs [3]. Physician-delivered preventative counselling such as advising patients to adopt a healthy diet and do more physical activities is an effective approach to reducing the prevalence of NCDs as it is applicable to both individual-and population-level health problems [4, 5].

As physicians in training, medical students will become the health care providers and have the responsibility to provide preventative counselling to their patients. Available research suggests that the healthy behaviors of physicians influence patients’ attitudes towards preventive counseling, in terms of their motivation to make healthy lifestyle choices [6]. Physicians who lead healthy lifestyles are more likely than others to advise patients to make healthy choices and lead healthy lives [7]. With this in mind, researchers have begun to realize the importance of studying the health status of medical students and how it relates to the delivery of preventive counselling. For example, Keller et al., found that the rate of binge drinking was 32 % among German medical students [8]. Furthermore, Chourdakis et al., found that 32.1 % of male and 8.4 % of female medical students were overweight in Greece [9]. A positive relationship between healthy lifestyles and attitudes toward preventative counseling was found in studies of medical students from the U.S. and Colombia [10–12].

To our knowledge, no study has been conducted to explore the differences in health-related behaviors and attitudes towards preventive counselling among medical students from different countries. Our study aimed to expand on previous findings by studying these differences between China, U.S. and Australia.

## Methods

### Ethic approval

This study was undertaken with ethic approval from the Peking University biomedical ethics committee, Duke University Institutional Review Board for Clinical Investigations and School of Population Health Research Ethics Committee at the University of Queensland.

### Study sample

Study subjects were recruited from five different Chinese medical schools, Duke University in U.S., and the University of Queensland in Australia. All those schools were chosen for convenience based on established collaboration within the network of the Center of Excellence at the George Institute. Chinese students were majoring in clinical medicine, enrolled in 2^nd^, 4^th^ and 7^th^ year at Peking University Health Science Center (PUHSC) in Beijing, School of Medicine, Xi’an Jiaotong University (XJTU) in Xi’an, China Medical University (CMU) in Shenyang, Ningxia Medical University (NMU) in Ningxia, and Changzhi Medical College (CMC) in Changzhi. American students were majoring in clinical medicine, enrolled in the Doctor of Medicine (MD) or MD/PhD (MSTP) program at Duke University, School of Medicine in Durham, North Carolina, U.S.. Australian students were majoring in clinical medicine, enrolled in 1^st^, 2^nd^, 3^rd^ or 4^th^year at the School of Medicine, the University of Queensland.

In terms of eligibility criteria, the participants had to have been enrolled before October 2012. This was because by the time the survey was administered, students enrolled by October 2012 would be in their 2^nd^ or greater year at their school. Students were considered eligible if they were enrolled in their 2^nd^, 4^th^, or 7^th^ year. These cohorts were selected to represent students from pre-clinical, new-clinical, and post-clinical populations from five different Chinese medical universities. The three cohorts of students began schooling in 2006, 2009 and 2011.

Based on a sample size calculation from the pilot study, the target sample size was 150 students from each grade, or 450 students in total, from the five medical universities in China. This was based on an estimate that 55 % students were considered to have positive attitudes towards preventive counselling from the pilot study (α = 0.05, absolute permissible error = 5 %, relative error = 10 % response rate = 70 %). The sample size was calculated as: N = U_(1-α)_^  2^/ Relative error^2^. We included all medical students from Duke University and from the University of Queensland. The total number of enrollments between October 2012 and November 2012 was 425 students from Duke University, 500 students from the University of Queensland, and 1568 students from the five Chinese medical schools. The non-response rates in the Duke University, University of Queensland, and five Chinese medical universities were 54.4, 50.5, and 79.2 %, respectively.

### Survey tool and distribution

This study examined the prevalence of healthy lifestyles among medical students and its association with attitudes towards preventive counseling of NCDs using a 32-item, self-reported online survey that took medical students approximately 15 min to complete.

The web-based survey consisted of four sections: 1. general health status; 2. personal health behaviors including physical activity, diet, mental health, smoking, alcohol consumption, health goals and health care seeking behaviors; 3. health care opinions and attitudes; 4. personal information. Many of the questions were adapted from validated sources, including the U.S. Centers for Disease Control (CDC) Behavioral Risk Factor Surveillance System (BRFSS) [13], CDC National Health Interview Survey [14], NHIS Tobacco Questions: 1997–2010 [15], and International Physical Activity Questionnaire (IPAQ) [16]. The inclusion of questions in the survey was based on consultations with the research team at the George Institute in China, a leader in the field of medical students’ lifestyles. The authors also referred to the study “Healthy Doctor = Healthy Patient”, by Professor Erica Frank from the University of British Columbia [17]. There were also questions that have been developed by the research team.

Before the formal implementation of the survey, we conducted a paper-based pilot study at Peking University Health Science Center in April 2012 to assess the acceptance of the questionnaire and made modifications as needed. Also to assess the feasibility of online survey and the response rate, an online pilot survey was administered to students at Peking University Health Science Center in September 2012 and further modifications were made as needed.

The final questionnaire was distributed in Chinese to Chinese medical students and in English to American and Australian medical students. Care was taken to ensure the consistence between the Chinese version and English version of the questionnaire. If the source of the questions were from validated English questionnaires, the questions were translated to Chinese and back translation was done to ensure the consistence of the meaning. And vice versa, if the source of questions were in Chinese, they were translated to English and back translation was done. Students were sent an email with a link to the questionnaire and were instructed to complete it within 2 weeks. In an additional effort to maximize the response rate, each medical school administrator followed up emails by sending four reminder emails, 4 weeks apart, between October 2012 and November 2012. Students who completed the survey received monetary compensation for their time and effort.

### The standard of healthy lifestyles (Exposure Variables)

The four domains of exposure variables were diet, exercise, smoking, and alcohol consumption. Those complying with dietary recommendations in the U.S. and Australia consumed more than five servings of vegetables and/or fruits per day [18]. In comparison, participants in China who reported consuming at least 200 grams of fruits and 300 grams of vegetables per day were considered to be in compliance with dietary recommendations [19, 20]. Participants who reported at least 150 min or more of moderate-to-vigorous physical activity per week were considered to be in compliance with exercise recommendations [21]. Participants were considered smokers if they reported having smoked at least 100 cigarettes in their lifetime and were currently still smoking cigarettes [22]. Alcohol consumption was divided into binge drinking and heavy drinking. Heavy drinkers were those who consumed, on average, at least one drink per day for women and at least two drinks per day for men in month [23]. Binge drinkers were those who reported drinking more than four drinks for women and more than five drinks for men in one sitting at any time during the past month. Participants who met dietary and exercise recommendations and who reported being non-smokers [15], non-heavy drinkers, and non-binge drinkers, were considered to have an overall healthy lifestyle.

### Outcome measures

The primary outcome measure of this study was the association between the healthy lifestyles and the attitudes towards preventive counselling.

### Statistical analysis

Univariate logistic regression analyses were performed to obtain an odds ratio (OR), 95 % confidence interval (95%CI) and *P*-value, to assess the association between healthy behaviors and attitudes towards preventive counseling among medical students at Duke University, the University of Queensland, and five medical schools in China. In addition, data was pooled according to country in order to compare findings from the U.S, Australia and China.

Differences in health behaviors and attitudes towards preventive counseling, due to level of education and level of professional development, may exist among students from different cohorts. Therefore, data was stratified by year to avoid effect modification. Prevalence estimates were obtained for all participants on self-reported body mass index, healthy behaviors (i.e., diet, exercise, smoking, and alcohol), and attitudes towards preventative counseling for each of these domains.

Multivariate logistic regression analyses were adjusted for gender to evaluate the association between healthy behaviors and attitudes towards preventative counseling between males and females. The main exposure variables (independent variables) were physical activity, balanced diet, weight control, stress management, smoking behavior and alcohol consumption. The main outcome variables (dependent variables) were students’ attitudes toward preventative counseling, which included basic knowledge of chronic diseases, stratified by year of training, country, and adjusted for gender; perceived adequacy of training; school’s promotion of each healthy habit; and so on. Collinearity between the domain variables was assessed by simple tabulation and any collinearity problems were noted in the tables. All analyses were performed using SPSS17.0 [24].

## Results

Self-reported health behaviors and attitudes towards preventive counseling among medical students are shown in Table 1. There are significant differences between self-reported health behaviors and attitudes towards preventive counselling for all participants, except for the following aspects: non-heavy or binge drinking (*χ*^2^ = 0.57, *p* = 0.75), attitude towards weight control (*χ*^2^ = 2.143, *p* = 0.342), attitude towards balanced diet (*χ*^2^ = 2.619, *p* = 0.27), and confidence to limit alcohol consumption (*χ*^2^ = 5.93, *p* = 0.052).

**Table 1:** Self-reported health behaviors and attitudes to preventive counseling among the medical students in the survey medical colleges (%)

	China	Duke	Queensland	Total	χ^2^	*P*
Reach the health standard						
18.5 < BMI < 25	1227/1552 (79.1)	162/228 (71.1)	129/183 (70.5)	1518/1963 (77.3)	12.65	0.002
Diet^a^	69/300 (23.0)	199/226 (88.1)	106/184 (57.6)	374/733 (51.0)	221.243	<0.001
Exercise^b^	481/1567 (30.7)	88/114 (77.2)	80/184 (43.5)	649/1865 (34.8)	108.039	<0.001
Non-smoking^c^	1447/1568 (92.3)	227/230 (98.7)	178/183 (97.3)	1852/1981 (93.5)	22.722	<0.001
Non-heavy or binge drink^d^	213/373 (57.1)	138/231 (59.7)	110/184 (59.8)	461/788 (58.5)	0.57	0.75
Stress^e^	1229/1568 (78.4)	162/231 (70.1)	130/184 (70.7)	1521/1983 (76.7)	11.823	0.003
Attitude						
Weight control	984/1568 (62.7)	120/231 (51.9)	95/184 (51.6)	1199/1983 (60.5)	2.143	0.342
Balance diet	1226 (78.2)	193 (82.5)	142 (77.2)	1561 (78.7)	2.619	0.27
Exercise	967 (61.7)	167/233 (72.8)	134 (72.8)	1268/1985 (63.9)	15.293	<0.001
Non-smoking	843 (53.8)	141 (61.0)	108 (58.7)	1092 (55.1)	64.215	<0.001
Limit alcohol	717 (45.7)	129 (55.8)	91 (49.5)	937 (47.3)	70.388	<0.001
Stress control	325 (20.7)	121 (51.7)	48 (26.1)	494 (24.9)	104.756	<0.001
Confidence						
Weight control	451/1568 (28.8)	82/233 (35.5)	68/184 (37.0)	601/1983 (30.3)	8.052	0.018
Diet	601 (38.3)	64 (27.5)	50 (27.2)	715/1983 (36.1)	17.518	<0.001
Exercise	584 (37.2)	113 (48.9)	89/114 (78.1)	786/1913 (40.1)	16.95	<0.001
Non-smoking	851 (54.3)	104 (45.0)	94 (51.1)	1049/1983 (52.9)	7.891	0.019
Limit alcohol	725 (46.2)	90 (39.0)	76 (41.3)	891/1983 (44.9)	5.93	0.052
Stress control	423 (27.0)	48 (20.8)	38 (20.7)	509/1983 (25.7)	7.098	0.029
Responsibility						
	1449/1478 (98.0)	217/234 (92.7)	177/185 (95.7)	1843/1897 (97.2)	22.16	<0.001
Education/Training						
Weight control	554/1568 (35.3)	137/233 (58.8)	154/182 (84.6)	845/1983 (42.6)	190.255	<0.001
Diet	784 (50.0)	120 (51.5)	129/185 (69.7)	1033 (52.1)	25.835	<0.001
Exercise	721 (46.0)	131 (56.2)	132 (71.4)	981 (49.5)	47.31	<0.001
Non-smoking	927 (59.1)	130 (55.8)	152 (82.2)	1209 (61.0)	39.752	<0.001
Limit alcohol	811 (51.7)	87 (37.3)	143 (77.3)	1041 (52.5)	67.457	<0.001
Stress control	592 (37.8)	84 (36.1)	92 (49.7)	768 (38.7)	10.768	0.005
School health environment^f^						
Encouraged healthy lifestyle	1505/1568 (96.0)	170/232 (73.3)	143/184 (73.3)	1818/1984 (91.63)	189.292	<0.001
Class of preventive medicine	1433 (91.4)	152 (65.5)	156 (84.3)	1741 (87.8)	127.651	<0.001
Good system to cope with stress	1116 (71.2)	194 (83.6)	117(63.2)	1427 (71.9)	23.041	<0.001
Encourages healthy eating healthy	1147 (92.3)	141 (60.8)	126 (68.1)	1414 (71.3)	227.76	<0.001
Encourage to exercise	1496 (95.4)	170 (73.3)	125 (67.6)	1791 (90.3)	231.051	<0.001
Discourages from smoking	1549 (98.8)	204 (87.9)	145 (78.4)	1898 (95.7)	201.506	<0.001

^a^Diet: Consuming ≥ 5 daily servings of fruits and/or vegetables (Duke and Queensland)

Consuming ≥ 200 g of fruits and ≥ 300 g vegetables daily (China)

^b^Engaging in ≥ 150 min/week of moderate-to-vigorous physical activity,

^c^Smokers are those who reported current smoking and having smoked ≥ 100 cigarettes in lifetime,

^d^Binge drinkers are those who consume ≥ 5 (males) or ≥ 4 (females) drinks of alcohol on a single occasion;

Heavy drinkers are males who drink on average > 2 drinks daily and females who drink on average > 1 drink daily

^e^Bear the stress during the past 2 weeks

^f^Students’ perception means the students that their school environment has encouraged them to lead healthy lifestyle

The results of the multivariate logistic regression analyses for all six health behaviors and attitudes towards preventive counseling are shown in Table 2. Male medical students’ were more positive in limiting their smoking behavior compared to female medical students (OR 1.7; 95 % CI 1.3–2.1). The unhealthy students were more passive (OR 1.7; 95 % CI 1.3–2.1) in weight control (OR 1.3; 95 % CI 1.1–1.7) and exercise (OR 1.7; 95 % CI 1.3–2.2) compared to healthy students. Students who reported greater confidence in providing preventive counselling service were more positive in weight control, exercise, and limiting smoking behavior (OR > 1) compared to students who reported less confidence. Students with good training were more positive in balance diet (OR 1.6; 95 % CI 1.1–2.4). Chinese medical students were more positive in stress control (OR > 1) and more passive in limiting their smoking and alcohol behaviors compared to medical students in Duke University. However, compared to medical students in University of Queensland, five Chinese medical students were more passive in stress control (OR < 1).

**Table 2:** The variables in the equation of multivariate logistic regression analyses in all six health behavior domains and attitudes towards preventive counseling^a^

Variables				95 % CI	
		Sig.	OR	Lower	Upper
Gender (female/male)	In weight control	<0.001	0.5	0.4	0.6
	In stress control	<0.001	0.4	0.3	0.5
	In limit smoking	<0.001	1.7	1.3	2.1
Behaviour (unhealthy/healthy)	In limit smoking	<0.001	0.3	0.1	0.4
	In limit alcohol	<0.001	0.5	0.3	0.7
	In stress control	<0.001	0.4	0.3	0.6
	In weight control	0.009	1.3	1.1	1.7
	In exercise	<0.001	1.7	1.3	2.2
Confidence (−/+)	In weight control	0.014	1.3	1.1	1.7
	In exercise	<0.001	1.6	1.3	2.1
	In limit smoking	0.010	1.4	1.1	1.9
Training (−/+)	In balance diet	0.017	1.6	1.1	2.4
Country					
(Duke/China)	In limit smoking	<0.001	0.1	0.0	0.2
	In limit alcohol	<0.001	0.3	0.2	0.5
	In stress control	0.034	1.5	1.0	2.2
(Queensland/China)	In stress control	<0.001	0.3	0.2	0.4

^a^Variable(s) entered on step 1: gender (female/male); behaviour; confidence; training; country (other country/China); about all six health behavior parameters: weight control/ exercise/ stress control/ limit smoking/ limit alcohol/ balance diet. Confidence to weight control, training in weight control, School health environment about weight control

Through the stratified multivariate logistic regression, differences between attitudes to preventive counseling and self-reported health behaviors are shown in Table 3. Medical students in senior grades (i.e., 3rd, 4th or 7th) who have more healthy behaviors have more positive attitudes towards preventive counseling for exercise (OR 1.9; 95 % CI 1.3–2.7) and weight control (OR 2.0; 95 % CI 1.4–2.7). Medical students in lower grades (i.e., 1st or 2nd) who have more healthy behaviors have more positive attitudes towards preventive counseling for exercise (OR 1.9; 95 % CI 1.4–2.6).

**Table 3:** The difference between attitudes to preventive counseling and self-reported health behaviors (Combined grade: 1 and 2 grade = the lower grade group, 3 and 4 or 7 = the senior grade group)

		Senior grade					Lower grade				
		Healthy	Unhealthy	χ^2^	*P*	OR (95 % CI)	Healthy	Unhealthy	χ^2^	*P*	OR (95 % CI)
Exercise	Attitude+	266 (83.9)	377 (73.2)	12.815	<0.001	1.9 (1.3, 2.7)	267 (80.4)	479 (68.3)	16.415	<0.001	1.9 (1.4, 2.6)
	Attitude-	51 (16.1)	138 (26.8)				65 (19.6)	222 (31.7)			
Weight control	Attitude+	438 (64.0)	89 (47.3)	17.189	<0.001	2.0 (1.4, 2.7)	500 (60.0)	154 (59.9)	0.007	0.995	1.0(0.8, 1.3)
	Attitude-	246 (36.0)	99 (52.7)				334 (40.0)	103 (40.1)			
Limit alcohol	Attitude+	63 (23.3)	37 (64.9)	38.327	<0.001	0.2 (0.1, 0.3)	79 (22.2)	58 (55.2)	42.398	<0.001	0.2 (0.1, 0.4)
	Attitude-	207 (76.7)	20 (35.1)				277 (77.8)	47 (44.8)			
Limit smoking	Attitude+	196 (23.6)	29 (55.8)	26.556	<0.001	0.2 (0.1, 0.4)	250 (24.4)	50 (64.9)	59.210	<0.001	0.2(0.1, 0.3)
	Attitude-	633 (76.4)	23 (44.2)				773 (75.6)	27 (35.10)			
Stress control	Attitude+	24(13.1)	182(20.6)	13.248	<0.001	0.4 (0.3, 0.7)	51 (18.2)	235 (28.6)	11.766	<0.001	0.6 (0.4, 0.8)
	Attitude-	158(86.9)	518(79.4)				229 (81.8)	586 (71.4)			
Diet	Attitude+	143 (83.1)	117 (79.1)	0.872	0.458	1.3 (0.7, 2.3)	154 (76.2)	147 (77.8)	0.131	0.972	0.9 (0.6, 1.5)
	Attitude-	29 (16.9)	31 (20.9)				48 (23.8)	42 (22.2)			

Through the stratified multivariate logistic regression, differences between attitudes and confidence to preventive counseling and self-reported health behaviors are shown in Table 4. Medical students in senior grades who have more confidence for preventive counseling, have more positive attitudes towards preventive counseling for weight control (OR 1.4; 95 % CI 1.0–1.9), balanced diet (OR1.4; 95 % CI 1.0–2.1), limit smoking (OR 1.5; 95 % CI 1.1–2.1), and limit alcohol (OR 1.6; 95 % CI 1.2–2.2). Medical students in lower grades (i.e., 1st or 2nd) who have more confidence for preventive counseling, have more positive attitudes towards preventive counseling for exercise (OR 1.7; 95 % CI 1.3–2.3), weight control (OR 1.4; 95 % CI 1.0–1.8), balanced diet (OR 1.6; 95 % CI 1.2–2.2), limit smoking (OR 1.4; 95 % CI 1.0–1.8), and limit alcohol (OR 1.4; 95 % CI 1.1–1.8).

**Table 4:** The difference between attitudes and confidence to preventive counseling and self-reported health behaviors (Combined grade: 1 and 2 grade = the lower grade group, 3 and 4 or 7 = the senior grade group)

		Senior grade					Lower grade				
		Confidence+	Confidence-	χ^2^	*P*	OR (95 % CI)	Confidence+	Confidence-	χ^2^	*P*	OR (95 % CI)
Exercise	Attitude+	86 (57.3)	366 (72.5)	12.398	<0.001	0.5 (0.4, 0.7)	323 (79.4)	481 (69.3)	13.162	<0.001	1.7 (1.3, 2.3)
	Attitude-	64 (42.7)	139 (27.5)				84 (20.6)	213 (30.7)			
Weight control	Attitude+	164 (65.9)	371 (58.6)	3.940	0.045	1.4 (1.0, 1.9)	228 (65.0)	432 (57.6)	5.390	0.017	1.4 (1.0, 1.8)
	Attitude-	85 (34.1)	262 (41.4)				123 (35.0)	318 (42.4)			
Limit alcohol	Attitude+	139 (37.0)	134 (26.5)	11.098	<0.001	1.6 (1.2, 2.2)	184 (35.7)	164 (28.0)	7.599	0.004	1.4 (1.1, 1.8)
	Attitude-	237 (63.0)	37 (73.5)				331 (64.3)	422 (72.0)			
Limit smoking	Attitude+	128 (30.0)	97 (21.5)	7.781	0.006	1.5 (1.1, 2.1)	184 (29.9)	116 (23.9)	5.013	0.023	1.4 (1.0, 1.8)
	Attitude-	303 (70.0)	354 (78.5)				431 (70.1)	370 (76.1)			
Stress control	Attitude+	66 (24.0)	140 (23.1)	0.085	0.943	1.1 (0.8, 1.5)	64 (27.6)	222 (25.5)	0.396	0.529	1.1 (0.8, 1.5)
	Attitude-	209 (76.0)	466 (76.9)				168 (72.4)	647 (74.5)			
Diet	Attitude+	296 (84.6)	421 (79.1)	4.102	0.036	1.4 (1.0, 2.1)	299 (81.9)	541 (73.5)	9.547	0.003	1.6 (1.2, 2.2)
	Attitude-	54 (15.4)	111(20.9)				66 (18.1)	195 (26.5)			

## Discussion

We studied the association between Chinese, American, and Australian medical students’ health behaviors and their attitudes towards preventive counseling. At present, the availability of data on medical students’ healthy behaviors and their attitudes towards preventive counseling in various countries is scarce. To determine the basic health behaviors of medical students in various countries, a survey was administered to medical students (*n* = 1983 individuals responding to the survey) from Duke University, the University of Queensland, and five Chinese medical universities, between October 2012 to December 2012. These universities were chosen as a convenience sample to be representative of international medical schools in geographic distribution. However, there is a limitation that there are considerable non-responses rates in the Duke University (45.6 %) and University of Queensland (49.5 %), while the non-response rate in five Chinese medical universities is relative low with 20.8 %. In our next research, we will choose more samples in order to guarantee the sample size in case of considerable non response rates. In addition, because of large sample of Chinese medical students, stratifying by grade was conducted. However, for U.S. and Australia medical students, this measure was not taken because the small enrollments. This is also a limitation of the study.

Research indicates that physician health can influence patients’ attitudes and motivation to make healthy lifestyle changes. Moreover, patients reported that physicians were more believable and motivating if they, themselves, practiced healthy lifestyles [10, 17]. The education of preventative behaviors provided to patients by physicians is necessarily key in helping patients to establish healthy lifestyles that reduce the likelihood of chronic diseases [4]. As physicians in training, much emphasis has been placed on the health behaviors of medical students. Frank administered a large-scale survey on health-related behaviors to medical students and other students in the United States. The results showed that medical students reported healthier lifestyles than non-medical students [25]. Boland conducted a longitudinal study found that Irish medical students reported increased alcohol consumption and decreased smoking in 1973, 1990 and 2002 [22]. A similar study in Greece found that medical students reported unhealthy behaviors, including heavy drinking, lack of exercise, and limited consumption of vegetables and fruit in their diet [9]. Tessier found that only 25 % of the medical students accepted the medical advice, in terms of healthy lifestyle choices, provided to patients. Furthermore, medical students with healthy lifestyles were easier to take the role, but male were more possible opposed to it [26]. A cross-sectional study in Colombia, using the questionnaire from “the Healthy Doctor = Healthy Patient”, found a positive association between medical students’ health habits and their attitudes towards preventive counselling [11]. These studies suggest that medical students should be guided to establish a healthy lifestyle so that they include preventive counselling more often in their practice and do it more effectively.

The findings from the current study are different to those reported by Frank in U.S. and Duperly in Columbia [11, 25]. Several reasons could explain the results. Firstly, we used a different method to examine attitudes towards preventive counselling. Previous studies directly examined as the extent to which students agreed or strongly agreed to use health communication to help patients change their lifestyles. In comparison, the current study adopted a more complex method which asked students to report not only responsibility to practice preventive counselling and training in preventive counselling, but also confidence to practice preventive counseling.

We examined students’ overall perception of the extent to which their school environment encouraged a healthy lifestyle. We were interested to compare findings in different countries and to examine the effect of school education on medical students’ healthy lifestyles and their future counselling practices. The findings indicate that students’ overall perception of the extent to which the school environment encouraged a healthy lifestyle was 96.0 % in Chinese universities, 73.3 % in Duke University and 76.5 % in The University of Queensland. These findings suggest that Chinese universities have more policies aimed at improving students’ lifestyle behaviors compared to the US and Australia. This may have a positive impact on Chinese physicians and their patients when it comes to preventive counseling. However, the system to cope with stress at Duke University was better than other universities. The overall results imply that medical schools encourage students to lead a healthy lifestyle through their health education programs and policies. This is key in helping medical students to form favorable attitudes towards preventive counseling.

The study found that medical students from lower grades had more positive attitudes towards preventive counselling for exercise, weight control, balanced diet, alcohol consumption, and smoking compared to students from senior grades. It is possible that students from lower grades have an exaggerated sense of enthusiasm towards preventive counseling for future patients. However, students from senior grades may have experienced difficulty with preventive counselling with patients in their clinical practice and built resistance to preventive counseling. Subsequently, senior medical students may have lost confidence to practice preventive counseling even if they had a healthy lifestyle with a balanced diet, adequate excise, and limited smoking. In our study, we also found that more than 92 % of students felt that they had a responsibility to provide preventive counseling to patients.

This study revealed that medical students with a healthy lifestyle were more likely to have a positive attitude towards preventive counselling and felt more confident in practicing preventive counseling, in terms of providing active and effective health dissemination advice. Medical institutions should aim to provide effective courses and training in health education in order to help medical students establish the ability and confidence to practice preventive counseling and to implement preventive strategies to reduce the likelihood of NCDs. In doing so, physicians can combine disease prevention and treatment strategies and interventions to lighten the burden of disease and save medical resources.

## Conclusion

1. Based on the finding that medical schools generally promote a healthy lifestyle, medical students have the encouragement and motivation from their respective universities to improve their health-related behaviors. Medical students at Duke University should improve non-heavy or binge drinking behaviors, medical students at The University of Queensland should improve exercise, weight control, and alcohol consumptions behaviors, and medical students at Chinese universities should improve diet, exercise, and alcohol consumption behaviors. In particular, Chinese students’ unhealthy diet and exercise behaviors are the most serious in all of the three countries. Based on findings, it seems that there is room for improvement in terms of a positive clinical practice attitude towards preventive counseling on all six health behavior domains.

2. The findings provide support for an association between self-reported health behaviors and attitudes towards preventive counseling. However, there are significant differences between countries in the association between attitudes towards preventive counselling and confidence to practice preventive counselling, even when adjusting for gender, a known predictor of positive clinical practice attitudes towards preventive counseling.

3. There is a need to provide training or courses in preventive counselling. While most medical students had positive attitudes towards preventive counseling, they lacked confidence, effective strategies, and the ability to practice preventive counseling.

4. The importance for medical students to lead healthy lifestyles should continue to be stressed in medical schools worldwide. As a result of schools’ improvement in promoting healthy lifestyles and positive clinical practice attitudes towards preventive counseling, there lies the potential for significant alleviations in the global burden of non-communicable disease.
